# Autologous Immune Cell-Based Regenerative Therapies to Treat Vasculogenic Erectile Dysfunction: Is the Immuno-Centric Revolution Ready for the Prime Time?

**DOI:** 10.3390/biomedicines10051091

**Published:** 2022-05-08

**Authors:** Michela Bonanni, Laura Rehak, Gianluca Massaro, Daniela Benedetto, Andrea Matteucci, Giulio Russo, Francesco Esperto, Massimo Federici, Alessandro Mauriello, Giuseppe Massimo Sangiorgi

**Affiliations:** 1 Department of Biomedicine and Prevention, Institute of Cardiology, University of Rome Tor Vergata, 00133 Rome, Italy; michelabonanni91@gmail.com (M.B.); gianluca88massaro@gmail.com (G.M.); dania.benedetto@gmail.com (D.B.); andrea.matteucci2@gmail.com (A.M.); giuliorusso.md@gmail.com (G.R.); 2 Athena Biomedical Innovations, 50126 Florence, Italy; laurarehak@gmail.com; 3 Division of Cardiology San Filippo Neri Hospital, 00135 Rome, Italy; 4 Department of Urology, University of Biocampus, 00128 Rome, Italy; francescoesperto@gmail.com; 5 Department of Systems Medicine, University of Rome Tor Vergata, 00133 Rome, Italy; federicm@uniroma2.it; 6 Department of Experimental Medicine, University of Rome Tor Vergata, 00133 Rome, Italy; alessandro.mauriello@uniroma2.it

**Keywords:** erectile dysfunction, cell therapy, stem cell, peripheral blood mononuclear cells, immune centric revolution, macrophages

## Abstract

About 35% of patients affected by erectile dysfunction (ED) do not respond to oral phosphodiesterase-5 inhibitors (PDE5i) and more severe vasculogenic refractory ED affects diabetic patients. Innovative approaches, such as regenerative therapies, including stem cell therapy (SCT) and platelet-rich plasma (PRP), are currently under investigation. Recent data point out that the regenerative capacity of stem cells is strongly influenced by local immune responses, with macrophages playing a pivotal role in the injury response and as a coordinator of tissue regeneration, suggesting that control of the immune response could be an appealing approach in regenerative medicine. A new generation of autologous cell therapy based on immune cells instead of stem cells, which could change regenerative medicine for good, is discussed. Increasing safety and efficacy data are coming from clinical trials using peripheral blood mononuclear cells to treat no-option critical limb ischemia and diabetic foot. In this review, ongoing phase 1/phase 2 stem cell clinical trials are discussed. In addition, we examine the mechanism of action and rationale, as well as propose a new generation of regenerative therapies, evolving from typical stem cell or growth factor to immune cell-based medicine, based on autologous peripheral blood mononuclear cells (PBMNC) concentrates for the treatment of ED.

## 1. Introduction

**Figure 1:**
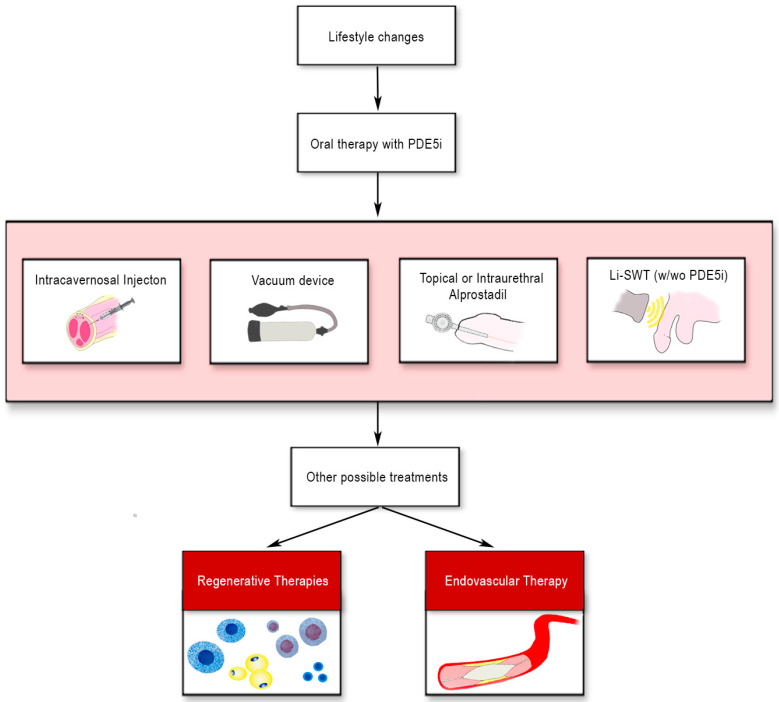
Current approaches to the treatment of erectile dysfunction.

Vasculogenic erectile dysfunction (ED) due to endothelial dysfunction and atherosclerosis of penile arteries is the most common cause of ED, especially in men over fifty [1]. According to the American Urology Association and the European Urology Association guidelines, the first-line treatment for vasculogenic ED consists initially of lifestyle changes, followed by oral phosphodiesterase 5 inhibitors (PDE5is) as first-line medical management [2,3]. However, about 35% of patients do not respond to oral PDE5i; on the other side, in patients who are responders, the compliance may be poor for the onset of side effects [4]. Moreover, in patients affected by diabetes mellitus, vasculogenic ED is more severe and refractory than in non-diabetic patients [5]. If PDE5i fails, second- and third-line treatment can be offered, including low-intensity shockwave therapy, intracavernosal injections therapy, vacuum erection devices, and intraurethral or topical application of prostaglandin E1 analogues, such as Alprostadil^®^ (Figure 1). 

However, even with the expansion of available treatments for ED, some patients still cannot achieve adequate performance. Surgical management with penile prosthesis implantation may be offered in patients who do not respond to second-line therapies [2]. In this setting, diabetic patients are more than twice as likely to undergo penile prosthesis surgery than non-diabetics [2,3]. Recently, alternative therapeutic strategies have been explored to treat non-responsive patients with vasculogenic ED effectively. 

**Figure 2:**
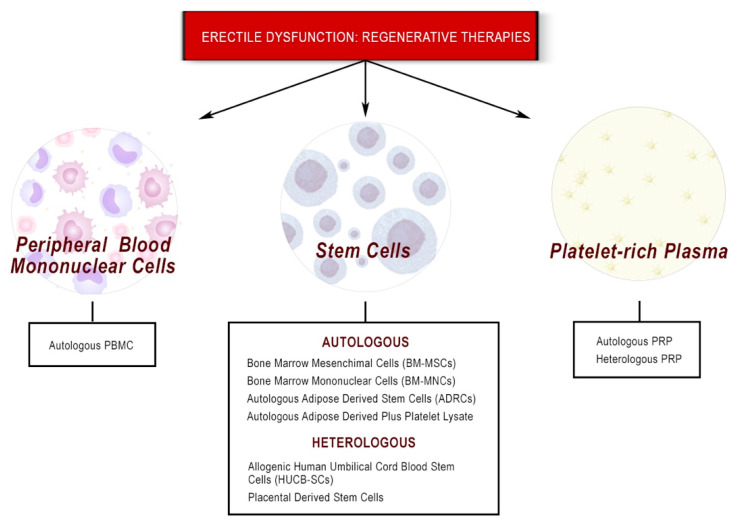
Summary of new regenerative medicine therapies available for the treatment of erectile dysfunction.

In this review, we provide an overview of cell-based regenerative therapies, including platelet-rich plasma (PRP), both heterologous and autologous stem cell therapy (SCT), and peripheral blood mononuclear cells (PBMNC), highlighting the role played by immune cell populations, which may represent the new frontier of vasculogenic erectile dysfunction treatment (Figure 2). 

## 2. Erectile Dysfunction in Patients Affected by Diabetes

Diabetes mellitus (DM) is one of the major traditional cardiovascular (CV) risk factors promoting vasculogenic ED, together with hypercholesterolemia, hypertension, and cigarette smoking [6]. According to multivariate analyses, DM determines the highest risk for ED among all CV risk factors [7,8]. It has been reported that the prevalence of ED in the diabetic population is about three times greater than that in non-diabetic individuals [9]. Among diabetic patients, ED is more common than retinopathy or nephropathy [10]. In 12% of type 1 diabetic men, ED was the first symptom of DM [11]. Moreover, ED also develops earlier in diabetic patients than in non-diabetic patients. In fact, within ten years of DM onset, more than 50% of patients develop ED [12]. 

The pathogenesis of diabetes erectile dysfunction (DED) is much more complex than in non-diabetic men. In fact, DM accelerates endothelial dysfunction and the atherosclerotic process through several alterations in molecular pathways, resulting in an inability to vasodilate the small penile arterioles. 

Decreased endothelial nitric oxide synthesis (eNOS) [13,14], selective degeneration of NO-dependent nitrergic nerves [15], increased advanced glycation end products (AGEs) and increased oxygen free radical content [16], decreased NO/cGMP signalling [17,18], increased endothelin B receptor binding sites [19], and an upregulated RhoA/Rho pathway [20] are just some of the mechanisms involved in the development of ED in diabetics. In addition to these known molecular pathways, NO mediates many of the antiatherogenic functions of the endothelium in patients with DED by blocking the expression of proinflammatory cytokines, chemokines, and leukocyte adhesion molecules [21]. Thus, loss of eNOS biological activity increases inflammation and cell proliferation. Indeed, increased expression of inflammatory markers was observed in these patients.

Moreover, AGEs themselves stimulate the expression of cytokines on monocytes and macrophages, while chronic hyperglycaemia leads to inflammation and contributes to the production of reactive oxygen species (ROS) [22,23]. In this context, the growing interest in the possible role of drugs that lower blood glucose levels, and thus chronic hyperglycaemia and AGEs, in erectile dysfunction is noteworthy. The recent review by Cignarelli et al. examined the mechanism of action of antidiabetic drugs in the possible remission of ED. Further studies are needed to define the role of these drugs in the treatment of ED [24].

It has been shown that circulating levels of endothelin-1 (ET-1) and cellular adhesion molecule-1 (ICAM-1), which are markers of inflammation, negatively correlated with the international index of erectile function score (IIEF). Moreover, circulating monocyte activity increased in patients with type 2 DM ED compared with type 2 DM without ED [25]. Thus, in patients with DED, all of the combined molecular mechanisms leading to endothelial dysfunction, together with associated inflammation, would appear to be the most common causes of nonresponse to PDE5i [26]. 

Several data suggested that men with DED have more severe and refractory ED and are more likely to be prescribed secondary ED treatments [27]. Therefore, an effective therapeutic strategy should simultaneously act on endothelial damage and inflammation. Moreover, limited blood flow in the cavernous bodies due to atherosclerotic plaques of the iliac-pudendal arterial vessels could be an additional cause of nonresponse to PDE5 inhibitors [28]. 

In this setting, percutaneous revascularization strategy may provide an alternative approach in those patients with refractory vasculogenic ED [29]. However, as with other second- and third-line approaches, endovascular strategy is also considered an invasive treatment. Regenerative cell therapies, on the other hand, are minimally invasive and can address the unmet medical need for alternative ED therapies that could restore natural erectile function.

## 3. Stem Cell Therapy and Erectile Dysfunction

Over the past few years, several studies have investigated the pre-clinical administration of stem cells (SCs) in animal models of erectile dysfunction. SCs are undifferentiated cells capable of unlimited proliferation, multi-differentiation potency, and perpetual self-renewal. The specific mechanisms underlying the effectiveness of SCs in the treatment of ED are not yet understood. Since pre-clinical studies have shown that few stem cells can be detected after transplantation, and almost no direct evidence supports the theory that transplanted stem cells have differentiated into vascular endothelial cells, smooth muscle cells, or nerves, the main mechanisms of action of stem cell transplantation would seem to be related to their paracrine action [30].

Moreover, preclinical research has shown that SCs exert their therapeutic effects on the basis of active factors contained in their secretions that can act as messengers. Indeed, the effect of SCs has been shown to persist after their disappearance, and even cell-free treatments have shown benefits [31,32]. Bioactive factors may represent a future treatment option for ED due to their pro-angiogenic, anti-inflammatory, anti-apoptotic, and anti-fibrotic properties [31,32]. 

**Figure 3:**
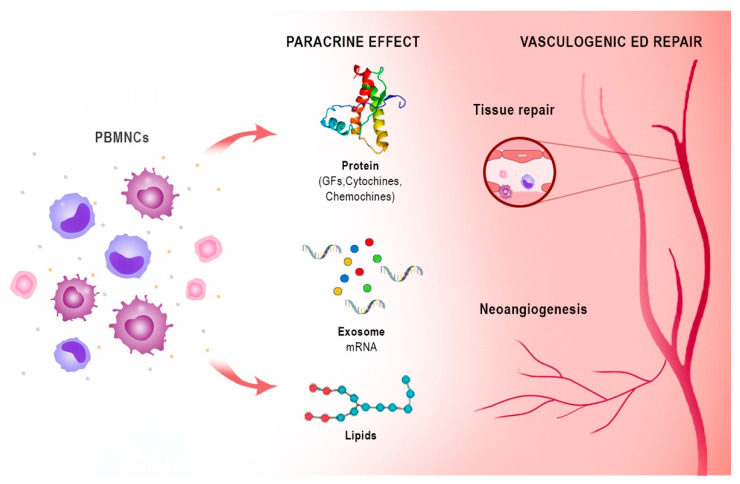
Immune-centric revolution: vasculogenic repair ED. PBMNCs: peripheral blood mononuclear cells. GFs: growth factors. PBMNCs based on monocytes/macrophages and lymphocytes are a novel autologous cell therapy that has shown efficacy in angiogenesis and tissue regeneration through the action of bioactive substances with paracrine effects represented by different types of protein molecules such as growth factors, chemokines and cytokines, lipid mediators as oxidized phospholipids, microRNAs, and exosomes.

Notably, the peripheral blood mononuclear cell (PBMNC) secretome differs only slightly from the stem cell secretome in its ability to promote cell proliferation [33]. These active paracrine factors, represented by different types of protein molecules, lipid mediators, microRNAs, and exosomes, underlie the regenerative effects of both stem cells and PBMNCs. The proteins are mainly growth factors, cytokines, and chemokines (e.g., CXCL8, CXCL5, CXCL1, CCL5, and VEGF). Lipids and especially oxidised phospholipids (e.g., PLPC-OOH, PAPC-OOH, SGPC, and PGPC) have also shown pleiotropic biological effects, such as neoangiogenesis, but also inflammation modulation by acting on Toll-like receptors (TLRs) and neutrophil granulocytes. Exosomes that arise intracellularly may contain a mixture of proteins, lipids, messenger RNA (mRNA), and micro RNA (miRNA). Due to the complexity of cellular paracrine activity, other factors also play an active role in this process, but these are the biological factors that have been most extensively studied both in vivo and in vitro [34]. Apoptotic PBMNCs have been shown to induce angiogenesis and vasodilation, enhance re-epithelialisation, promote macrophage polarization, and modulate the immune system through their paracrine factors. Since the isolation and cultivation of stem cells is not easy, while the secretome is easier to obtain, the latter could take on a central role in regenerative therapy [34] (Figure 3).

In acute disease models, such as cavernous nerve injury ED, paracrine mechanisms seem to be the primary mechanism of the SCs’ action [29]. There is no recognizable and temporally defined acute injury in chronic ED models, including aging, diabetes mellitus, and hyperlipidaemia. The mechanisms underlying established ED act at several levels, leading to different cell types of damage. Therefore, the SCs therapy is much harder to investigate in chronic disease models and should focus on nerve restoration and vascular recovery. 

The efficacy of SCs treatment was initially evaluated in ED with acute cavernous nerve injury (CNI) [30]. Subsequent studies have assessed the effects of SCs treatment in chronic injury ED in diabetic patients. Garcia et al. [35] first identified the impact of autologous adipose-derived stem cells (ADSCs) on rats with DM. In 2016, Li et al. performed a meta-analysis on ten pre-clinical studies that used 302 rats to determine the optimal SCs therapeutic strategy for DED 

Pooled analysis showed a positive effect of SCs therapy on improving erectile function in diabetic rats. In the SC therapy group, the smooth muscle and endothelial cell content was significantly higher than in the control group [36].

Finally, recent studies have evaluated the use of SCs therapy as a treatment for ED in humans. The most studied SC types in ED include allogeneic human umbilical cord blood stem cells (HUCB-SCs), placental matrix-derived mesenchymal stem cells (PL-MSCs), ADSCs, and bone marrow mononuclear cells (BM-MNCs).

### 3.1. Heterologous Stem Cell Therapies: Cord Blood/Placenta Derived

Bahk et al. [37] reported the results of a single intracavernous infusion of HUCB-SCs into the corpora cavernosa of seven diabetic patients with ED. HUCB-SCs, in a total cell number of around 1.5 × 10^7^, were injected into both corpus cavernosa of each patient. Outcomes were assessed using the Index of Erectile Function-5 (IIEF-5) score, the Sexual Encounter Profile (SEP), Global Assessment Questionnaire (GAQ), erection diary, and blood glucose. Patients were followed for 11 months. They reported a simultaneous improvement in the libido, ED, and blood glucose levels after transplant of HUCG-SCs, compared to the control group. 

In 2016, Levy et al. [38] determined the feasibility and effects of using single injection PL-MSCs to treat eight patients with chronic, organic ED. Outcomes were evaluated with doppler parameters and erectile function questionnaires. The results indicate that this treatment may be beneficial, with a significant increase in the peak systolic velocity (PSV) at six weeks, three months, and six months follow-up. Conversely, the end-diastolic velocity and IIEF score were not statistically significant.

### 3.2. Autologous Stem Cell Therapies: Bone Marrow and Adipose Tissue

In 2016, Yiou et al. studied twelve animal models that mimic erectile dysfunction after radical prostatectomy and were treated with increasing numbers of BM-MNCs. After six months, erectile function and penile vascularisation improved significantly. Patients who received higher doses showed a much more significant improvement in spontaneous erections. The clinical benefits were related to improvements in PSV and penile release NO. These benefits persisted after one year. After a mean follow-up of 62.1 ± 11.7 months, erectile function scores were lower than at 1-year follow-up. It was concluded that repeated intracavernosal injections of BM-MNCs are necessary to prevent a gradual decline in erectile function over time [39,40].

In 2018, an open-label phase I clinical trial [41] was conducted to evaluate the safety and efficacy of autologous bone marrow-derived mesenchymal cells (BM-MSCs) in the treatment of four diabetic patients with refractory DED. All patients received two consecutive intracavernosal injections of autologous BM-MSCs at baseline and 30 days later. Safety and tolerability were the primary outcomes of the study, while the secondary outcome was to evaluate the efficacy of the procedure, as assessed by the International Index of Erectile Function-15 (IIEF-15) and the Erection Hardness Score (EHS). The results indicate that the intracavernosal BM-MSC injections were well tolerated as no patient reported significant adverse effects and the sexual function scores improved significantly.

In 2020, Bieri et al. [42] investigated the injection of autologous bone marrow concentrate (Caverstem 1.0 low dose—Caverstem 1.0 high dose—Caverstem 2.0) in patients with vascular origin ED, who did not respond to phosphodiesterase-5 inhibitors. There was an improvement in the mean IIEF-5 score in all three groups: two in the low-dose Caverstem 1.0 group, three in the high-dose Caverstem 1.0 group, and nine in the Caverstem 2.0 group. These results were maintained at the six-month follow-up. There was no statically significant change in PSV in the low- or high-dose group.

In 2018, Haahr et al. [43] reported on a phase 1, 1-year, prospective clinical trial conducted to assess the safety and potential effect of a single intracavernous injection of autologous ADSCs in 21 men with refractory ED following radical prostatectomy. Six men were incontinent, and fifteen were continent at inclusion. All patients received a single intracavernous injection of ADSCs and were followed for one year. Erectile function was assessed by the IIEF-5 score and EHS. Eight reversible minor events were reported. Eight patients recovered their erectile function and regained the ability to perform sexual intercourse. Moreover, their IIEF-5 score continued to improve throughout the 12-month study. However, incontinent patients reported no significant improvements in erectile function, and their IIEF-5 score and EHS score that did not significantly differ from those registered at the time of inclusion into the study. 

In 2019, Protogerou et al. [44] evaluated the safety and efficacy of ADMSCs and platelet lysate (PL) in organic ED. ADMSCs resuspended in PL were administered to five patients, while three patients were treated with PL alone. No major adverse reactions occurred. Improvement in the IIEF-5 score was seen in both patient groups at both 1 month and 3 months after treatment. 

In 2020, the authors presented the results after six months of Group A who received stem cells and PLP. The IIEF-5 score and PSV improved in all patients. Results regarding End Diastolic Velocity (EDV) were more variable. No side effects were noted. Therefore, the authors concluded that stem cell therapy in combination with PL appears to improve erectile function and has minimal side effects in the short term [45]. 

Some critical issues on autologous cell therapy in diabetic patients should be considered. Diabetes could heavily impair the angiogenic and regenerative capacity of autologous cell therapy [46]. It is well known that diabetes induces a deficiency in vascular regenerative cells and angiogenic capacity, confirmed by an increased risk of cardiovascular diseases in these patients. In particular, diabetes causes an extensively documented functional bone marrow impairment [47]. Bone Marrow stem cells CD34+ from diabetic patients do not respond to hypoxia and show a reduced paracrine release together with a diminished angiogenic potential [48]. 

These data demonstrate a reduced angiogenic capacity of bone marrow-derived cells in diabetic patients and are consistent with the randomised MOBILE trial conducted in 155 patients with critical limb ischaemia, in which the one-year rate of amputation-free survival of patients receiving BM-MNCs compared with placebo was not significant. In addition, a two-year post hoc analysis showed that BM-MNCs provided significant benefit for patients without diabetes at Rutherford stage 4, but no benefit for diabetic patients and Rutherford stage 5 patients [49].

In contrast, the study by Lu et al., who enrolled forty-one diabetic patients with critical limb ischemia, suggested that treatment with BMMSC promotes limb blood flow and ulcer healing, and reduces ulcer recurrence and amputation within 9 months. The actual benefit of regenerative stem cell therapies in diabetics therefore remains to be investigated [50].

Moreover, diabetes has been demonstrated to impair adipose tissue-derived stem cell wound healing ability [51]. A substantial reduction in VEGF (Vascular Endothelial Growth Factor) secretion and an impaired angiogenic capacity have been shown in ADSCs from diabetic patients [52,53]. These data agree with a recent comparison of the secretomes released from adipose tissue-derived cells, bone marrow, and umbilical cord Wharton’s jelly: umbilical cord secretomes showed a complete angiogenic complex with higher angiogenesis proteins, followed by bone marrow secretomes [54]. ADSC secretomes, instead, missed the essential angiogenic proteins and expressed most angiogenic proteins to a significantly reduced level [53]. Some authors recently suggested that a structural dysfunction of mesenchymal stem cells isolated from diabetic patients may limit their potential therapeutic use [55].

In conclusion, stem-cell therapy was considered a promising therapeutic option for ED patients; however, future studies are needed to define the best tissue source, safety and efficacy profile, dosage, and the exact mechanism of action, particularly in chronic conditions of organic and diabetic ED. Currently, there are 22 trials listed on the ClinicalTrials.gov website regarding the treatment of ED. Among these trials, seven have been withdrawn or suspended for various causes, such as insufficient recruitment, lack of responses, and lack of funding. Only nine trials are active or have been completed (NCT02945462, NCT02344849, NCT02945449, NCT03751735, NCT02398370, NCT01089387, NCT03699943, NCT01953523, and NCT02472431). Stem cell therapy for ED has only been studied in a limited capacity. Each study so far has its customized protocol and there are no standardized protocols for cell therapy in the treatment of ED. Optimization of cellular preparation and development of a standardized method for the cells’ application in terms of cell type, cell number, application, point of care system, or GMP cell expanded production and outcome measures are suggested.

Moreover, cost, source, simplicity of isolation and culture, risks, and effectiveness must be considered when selecting the most appropriate type of regenerative cell-based therapy. There is a definite need for more extensive, placebo-controlled, double-blinded, and randomized trials to overcome all significant biases. Both the European Association of Urology (EAU) and Sexual Medicine Society of North America (SMSNA) recognize the therapeutic potential of autologous cells therapy while also stating that stem cells require further investigation in large-scale randomized clinical trials before they are included as recommended modalities for ED treatment.

## 4. Platelet-Rich Plasma Therapy

Although PRP is an emerging treatment option in several fields of medicine, including ED, there are limited studies that support its efficacy. Ding et al. [56] studied the PRP effect on regeneration and restoring the cavernous nerve function after its damage in a rat model. Animals were divided into three groups receiving either sham operation, bilateral cavernous nerve (CN) crush injury with immediate injection of PRP at the site of injury, or bilateral CN crush injury with no further intervention. Intracavernous pressure (ICP) was measured to evaluate erectile function. In the PRP group, the ICP was significantly higher than in the non-treated group but lower than in the placebo group (*p* < 0.05). 

Furthermore, in the PRP treatment group compared to the other injured group, they found more CN myelinated axons and more NADPH-diaphorase-positive nerve fibres.

In 2012, Wu et al. [57] reported that rats in the experimental group receiving intralesional PRP therapy immediately after the CN damage improved erectile function (*p* < 0.05) and improved preservation of myelinated axons of the CN significantly (*p* < 0.05) compared with animals that did not receive PRP therapy. PRP administration significantly reduced the level of apoptotic markers, as a reduction in TGF-b1 staining. The absence of the type III collagen and the prevalence of the type I collagen was observed in the histologic study of penile tissue from the treated group. The authors believe that PRP accelerated the nerve repair processes through its neuro-regenerative and neuroprotective effects, and it also inhibited the fibrosis process in the corpora cavernosa. The limit of the study was the small sample of rats; this did not allow us to consider the use of PRP therapy as an effective treatment method, despite the positive results. 

Another experimental study by Wu et al. [58] was conducted with the aim of comparing the effects of PRP from different preparation methods on the restoration of erectile function in a rat model. In this study, rats were randomly divided into four groups: the first group received sham operation, and the other three groups received bilateral CNS crush injury. The last three groups were treated with PRP, PRP with an increased PDGF-AB concentration, or normal saline injected into the corpora cavernosa at the time of injury. The authors concluded that PRP, which was optimised with high levels of growth factors, was more stable and promoted recovery of erectile function.

Recently, Epifanova et al. [59] conducted a clinical trial to evaluate the safety and efficacy of PRP in the treatment of ED. Patients were randomly divided into three groups: 30 patients who received an intralesional injection of PRP activated with a 10% CaCl2 solution, 30 patients who received an intralesional injection of PRP activated with a 10% CaCl2 solution plus PDE5i, and 15 patients who received inactivated PRP. The patients received three PRP injections at weekly intervals. At the end of treatment, the authors assessed erectile function by analysing PSV, resistance index, IIEF-5 score, and SEP score. Although the results showed improvements in erectile function in all three groups, the study is limited by the lack of comparison with placebo and long-term effects. It was therefore concluded that PRP is a safe therapy and contains sufficient levels of growth factors to achieve the therapeutic effect.

Matz et al. [60] conducted a retrospective study of 17 patients to evaluate the safety and feasibility of platelet-rich fibrin matrix (PRFM) for treating urologic conditions, such as ED. Patients received 1–8 injections of 4–9 mL of PRFM. The IIEF-5 scores were reviewed before and after injections. The IIEF-5 score for men increased on average by 4.14 points. Post-procedural minor adverse events were seen in three men. The authors concluded that PRFM injection is a safe and feasible treatment modality and could possibly treat ED. However, evaluation of effectiveness would require objective study methods rather than just questionnaires.

In conclusion, although it seems to be a promising therapy, more extensive studies are needed to understand PRP’s mechanism of action better before PRP therapy enters clinical practice.

## 5. Harnessing the Immune System for Tissue Repair and Regeneration: A Lesson from the Heart

Tissue healing and regeneration is a complex, organized, spatiotemporal process involving a plethora of cell subsets, the action of which is strictly regulated to obtain an effective tissue [61]. Heart disease, critical limb ischemia, diabetic foot, and severe musculoskeletal disorders require new therapeutic strategies to repair damaged tissue, especially considering an aging population where diabetes and obesity have reached gigantic proportions.

Clinical trials using adult stem cells to regenerate damaged heart tissue are ongoing, notwithstanding the questions of efficacy and scarcity of understanding of the mechanism of action and of the biological effects [62]. The rationale for adult stem cell therapy clinical trials to repair damaged heart tissue is derived from animal studies that showed a limited but reproducible recovery in cardiac function after ischaemic injury [63]. Vagnozzi and co-authors proved that after cells implant after ischaemia–reperfusion injury, although heart function was improved, it was not correlated with the production of new cardiomyocytes [64]. On the contrary, two different types of adult stem cell bone marrow mononuclear cells (BM-MNCs), which were the most heavily used stem cell type used in clinical trials, and cardiac mesenchymal cells from the heart that express the receptor tyrosine kinase c-Kit), improved heart function through an acute sterile immune response due to strong recruitment of specific macrophage populations (CCR2+ and CX3CR1+). Moreover, both intracardiac injection of killed stem cell or zymosan, a non-cellular and potent activator of the innate immune response, induced an analogous local macrophage accumulation that provided functional recovery after ischemic damage. Vagnozzi et al. proved that this selective macrophage response acted in multiple fashions altering the activity of the cardiac fibroblasts, reducing in the border zone the extracellular matrix content, and increasing the mechanical asset of the damaged area. 

These data showed that cardiac cell therapy’s functional benefit was due to the immune system’s acute inflammatory wound-healing response. Interesting to notice, and surprisingly, a zymosan immune-based response maintained its effect for a longer time as opposed to stem-cell therapies. 

By suppressing macrophage activity in some rats, the researchers showed that the repair process did not occur in either stem cell-implanted or zymosan-treated rats, highlighting the importance of the immune system. This study shows that the repair mechanism is driven by the immune system and that the results could also apply to stem cell therapies used for other diseases. Therefore, the reparative process triggered by stem cells probably derives from an acute immune response rather than from the regenerative capacity of the cells themselves [65]. Accordingly, Godwin et al. have shown in an ischaemic animal model where depletion of macrophages leads to complete failure of regeneration despite proliferation of cardiomyocytes [66].

Consistent with these observations, Mordechai et al. [67] in 2013 and Pinto et al. [68] in 2014 demonstrated that macrophages regulate resident stem cells and promote myocardial tissue regeneration after ischaemia. Therefore, a new strategy to improve infarct healing may be to target the macrophages themselves. Similarly, Navarro et al. in 2014 showed that the main agents responsible for tissue vascularisation and angiogenesis are the monocytes resident in the adipose tissue, compared to the stromal vascular fraction-derived MSCs [69].

The authors observed that neovascularization of the implants containing SVF monocytes or PB monocytes was 3.5 or 2 times higher than that observed in the implants with SVF-derived MSCs in a quantitative analysis of angiogenesis at 14 days after cell implant. Moreover, Cai et al. [70] observed that early depletion of macrophages in adipose tissue resulted in incompetent angiogenesis, reduced stem cell recruitment, and a poor retention rate. In contrast, upregulated macrophages allowed better angiogenesis and survival, suggesting that macrophages are closely associated with tissue regeneration.

The predominant role of the immune system in stem cell-driven regeneration has also been suggested by studies conducted in various tissues, such as bone regeneration [71], knee osteoarthritis [72], cervical spine fusion [73], muscle regeneration [74], and cartilage repair [75,76].

Another reason that might change the focus away from stem-cell therapies is that they are expensive to generate and require time-consuming approval from regulatory agencies. This change in paradigm, which has been called an immune-centric revolution or macrophage-centred approach, suggests the opportunity to focus on the role of immune cells in the body rather than on stem cells and could be applied to different conditions ranging from critical limb ischemia, wound healing, and musculoskeletal disease to even ED [77,78,79,80].

## 6. Harness Peripheral Blood Mononuclear Cells Angiogenic Potency: From Critical Limb Ischemia to ED

PBMNCs based on monocytes/macrophages and lymphocytes are an innovative autologous cell therapy that have shown angiogenesis potency and tissue regeneration in no-option critical limb patients and in diabetic foot patients [80,81,82,83] (Figure 3). The detailed mechanism of action of PBMNCs is beyond the scope of this review and is adequately described in a review on autologous cell therapy [46]. Briefly, the angiogenic and arteriogenic ability of monocytes/macrophages is well known and extensively described [84,85,86,87].

It has also been observed that monocytes/macrophages are able to repair cerebrovascular ruptures in haemorrhagic strokes due to their ability to physically adhere to rupture sites and generate mechanical traction [88]. Krishnasamy et al. demonstrated that Notch signalling recruits macrophage differentiation and maturation from monocytes, promoting arteriogenesis and tissue repair in ischaemic tissue [89].

It is important to note that monocytes also maintain angiogenic potency in diabetic patients, while hematopoietic stem cells do not [48,90]. Thus, in diabetic patients who show a compromised CD34+ cells population, monocytes (CD14++ cells) can provide an alternative therapeutic option. Lymphocytes and monocytes have been observed to have a critical role in angiogenesis [91,92]. In particular, regulatory T cells (Tregs), a specific subpopulation of lymphocytes, are crucial mediators of immune homeostasis, regulating the immune response by suppressing inflammation and promoting self-tolerance. In addition, a growing body of evidence points out a critical role of Tregs in angiogenesis [93,94]. Moreover, it has been observed in an ischemic animal model that a lack of lymphocytes impairs both macrophage polarization, which is essential for tissue regeneration, and angiogenesis in diabetic wound healing [95]. 

In addition, Leung et al. have shown that in ischemic tissue in diabetics, when Th1 function is impaired, Tregs promote post-ischaemic revascularization [96].

Indeed, while lymphocyte Th1 cells impair vascular regeneration in diabetic patients in a paracrine manner, Tregs potentiate regeneration. The numerous scientific pieces of evidence, in vitro and in vivo in animal models, indicating the robust angiogenic capacity of peripheral blood mononuclear cells, both monocytes/macrophages and lymphocytes, are confirmed in many clinical trials in the treatment of critical limb ischemia and the diabetic foot. 

In a recent meta-analysis, Rigato et al. analysed 19 randomised controlled trials, 7 non-randomised trials, and 41 uncontrolled trials investigating the efficacy of autologous cell therapy for intractable critical limb ischaemia. It was found that PBMNCs, but not BM-MNCs or BM-MSCs, were associated with a significant decrease in amputations and an increase in amputation-free survival, that intramuscular implantation appears to be more effective than intra-arterial infusion, and that peripheral blood mononuclear cells can outperform mesenchymal and bone marrow stem cells. Using hierarchical meta-regression, the authors found that in studies with a higher prevalence of diabetic patients, the benefit of cell therapy on amputation rates was higher. [97]. In conclusion, the authors suggest that autologous cell therapy has potential for the treatment of intractable critical limb ischaemia, although more high-quality placebo-controlled trials are needed [97].

A point-of-care (POC) device has recently shown reasonable efficacy in therapeutic angiogenesis both in vivo and in vitro. It is a device based on selective filtration of peripheral blood and is intended for intraoperative use in human cell therapy to produce fresh autologous PBMNCs. [97,98]. PBMNCs produced with this device (Hematrate Blood Filtration system—Cook Regentec) have shown promising results in several clinical trials, including studies in diabetic patients [81,99,100]. Persiani et al. [82] observed the effects of PBMNC therapy in 18 non-option patients with diabetes and critical limb ischaemia and found an increase in the mean transcutaneous partial pressure of oxygen (TCPo2) and a reduction in pain after two years.

Recently, Scatena et al. [101] showed that PBMNCs manifested a favourable clinical outcome at two years follow-up in patients with diabetic foot and critical limb ischemia not feasible of revascularization, which significantly reduced the amputation rate and improved survival wound healing. Trophic support of the neo-endothelium is provided by bioactive and angiogenic factors produced by PBMNCs that act in a paracrine manner [86,102]. 

On these bases, our group studied the treatment of a young diabetic patient that suffered from severe vasculogenic ED who did not respond for more than one year to therapy with oral PDE5i and intracavernous PDE1 [103]. After detection of severe atherosclerotic disease of the internal iliac artery and pudendal on selective angiography of the pelvic district, the patient was treated by revascularization with a drug-coated balloon and drug-eluting stent placement combined with multiple intra-cavernous injection of autologous mononuclear cells from peripheral blood [81]. At six months, dynamic Doppler ultrasound together with the IEF-5 score showed an excellent mid-term result. At one year, the patient reported a stable improvement in sexual function. This procedure is quick and easy to perform; PBMNCs are produced in a closed disposable POC, concentrated with minimal manipulation and injected in the same surgical procedure. In addition, blood sampling is non-invasive, and therapy can be repeated. The frequency of implantation rather than the absolute number of implanted cells appears to be critical [104,105]. A larger case series is currently being actively enrolled to confirm this preliminary experience.

## 7. Conclusions

Currently, recommended ED treatments frequently do not achieve adequate results, particularly in diabetic patients. Regenerative therapies, including platelet-rich plasma (PRP) and stem cell therapy (SCT), are starting to be used for ED treatment as an adjunct or alternative therapy, although on a limited number of patients. PRP delivers an autologous sample rich in growth factors to damaged tissue. PRP studies have shown an increased erectile function recovery and preservation of cavernous nerve axons on animal models; however, studies with PRP in humans are very limited. SCT has been used in diabetic patients and post-prostatectomy ED with mixed results in clinical trials, although SCT treatments improved erectile rigidity and functionality. Still, there is a lack of evidence to support the efficacy of these treatments. The scenario seems similar to the initial enthusiasm for cell therapy in ischemic heart disease, which was dampened by less than brilliant results in the clinic despite promising efficacy data in animal models.

In addition to stem cells and growth factors, the immune system plays a crucial role in tissue healing. PBMNCs showed a robust rationale and extensive clinical data, particularly for treating critical limb ischemia and diabetic patients. Their effect is correlated to a dual mechanism of action based on angiogenesis and macrophage polarization, which is essential in tissue regeneration. We believe it is mandatory to exploit the healing resources of these immune cell types. Immune cell-based therapy and PBMNC cell therapy require further investigation in large-scale randomized clinical trials before they are included as recommended modalities for ED treatment.

## Data Availability

Not applicable.
